# Sequential pattern mining for discovering gene interactions and their contextual information from biomedical texts

**DOI:** 10.1186/s13326-015-0023-3

**Published:** 2015-05-18

**Authors:** Peggy Cellier, Thierry Charnois, Marc Plantevit, Christophe Rigotti, Bruno Crémilleux, Olivier Gandrillon, Jiří Kléma, Jean-Luc Manguin

**Affiliations:** INSA de Rennes, IRISA, UMR6074, Rennes, F-35042 France; Université de Paris 13, LIPN, UMR7030, Villetaneuse, F-93430 France; Université Lyon 1, LIRIS, UMR5205, Lyon, F-69622 France; INSA de Lyon, LIRIS, UMR5205, Lyon, F-69621 France; Université de Caen, GREYC, UMR6072, Caen, F-14032 France; Université Lyon 1, CGMC, UMR5534, Lyon, F-69622 France; Faculty of Electrical Engineering, Czech Technical University, Prague, Czech Republic

**Keywords:** Data mining, Sequential pattern mining, Natural language processing, Information extraction, Gene interactions

## Abstract

**Background:**

Discovering gene interactions and their characterizations from biological text collections is a crucial issue in bioinformatics. Indeed, text collections are large and it is very difficult for biologists to fully take benefit from this amount of knowledge. Natural Language Processing (NLP) methods have been applied to extract background knowledge from biomedical texts. Some of existing NLP approaches are based on handcrafted rules and thus are time consuming and often devoted to a specific corpus. Machine learning based NLP methods, give good results but generate outcomes that are not really understandable by a user.

**Results:**

We take advantage of an hybridization of data mining and natural language processing to propose an original symbolic method to automatically produce patterns conveying gene interactions and their characterizations. Therefore, our method not only allows gene interactions but also semantics information on the extracted interactions (e.g., modalities, biological contexts, interaction types) to be detected. Only limited resource is required: the text collection that is used as a training corpus. Our approach gives results comparable to the results given by state-of-the-art methods and is even better for the gene interaction detection in AIMed.

**Conclusions:**

Experiments show how our approach enables to discover interactions and their characterizations. To the best of our knowledge, there is few methods that automatically extract the interactions and also associated semantics information. The extracted gene interactions from PubMed are available through a simple web interface at https://bingotexte.greyc.fr/. The software is available at https://bingo2.greyc.fr/?q=node/22.

## Introduction

Literature on biology and medicine represents a huge amount of knowledge: more than 24 million publications are currently listed in the PubMed repository [1]. These text collections are large and it is difficult for biologists to fully take benefit from this incredible amount of knowledge. A critical challenge is then to extract relevant and useful knowledge spread in such collections. Text mining and Natural Language Processing (NLP) are rapidly becoming an essential component of various bio-applications. These techniques have widely been applied to extract and exploit background knowledge from biomedical texts.

Among many tasks, a crucial issue is the annotation of a large amount of genetic information. NLP, and Information Extraction (IE) in particular, aim to provide accurate processing to extract specific knowledge such as named entities (e.g., gene, protein) and relationships between the recognized entities (e.g., gene-gene interactions, biological functions). Databases such as BioGRID [2] or STRING [3] store a large collection of interactions derived from different sources and indicate which gene interacts with a specified gene. However, these databases do not support more complex requests such as: *which genes inhibit gene X?what is the biological context (e.g., organism, biological information) associated to a gene-gene interaction?what is the kind of interaction between genes X and Y?what is the modality associated to the extracted information (related work, experimental result, etc.)?* These requests are useful for biologists since they enable to faster point out the piece of information they look for. Unfortunately, to the best of our knowledge, no work has been reported yet to support these kinds of requests. That is why in this paper we propose a method to retrieve that kind of information.

Our method automatically discovered a human manageable set of patterns that are then validated by experts to provide linguistic patterns. In other words, thanks to the linguistic patterns, our method not only allows gene interactions but also *semantics information on the extracted interactions (e.g., modalities, biological contexts, interaction types)* to be detected.

The need for linguistic resources (grammars or linguistic rules) is a common feature of the information extraction methods. Indeed, those NLP approaches apply rules such as regular expressions [4] or syntactic patterns [5,6]. However, these rules are handcrafted and thus those methods are time consuming and often devoted to a specific corpus [7].

In contrast, machine learning based methods, for example support vector machines or conditional random fields [8], are less time consuming than rule-based methods. Machine learning methods for gene interaction detection usually tackle the task as a classification problem. Best results are obtained with kernel methods [9-12] and some NLP parsers can be used to provide some features to the classifier [13]. Although they provide good results, machine learning methods still need many features. Also, their outcomes are not really understandable by a user, nor they can be used as linguistic patterns in NLP systems. Furthermore, the annotation process of training corpora requires a substantial investment of time, and cannot be reused in other domains (some new corpora must be annotated for new domains) [7]. A good trade-off is the cross-fertilization of information extraction and machine learning techniques which aims at automatically learning the linguistic rules [14,15]. However, in most cases the learning process is done from text syntactic parsing. For instance, BioContextt [16] or Turku Event Extraction System (TEES) [17] aim at extracting biological events with contextual informations (e.g., species involved, localization, modality) about the biological events. Those systems are based on a syntactic analysis. Therefore, the quality of the learned rules relies on syntactic process results. Still some works such as [18] or [19] do not use syntactic parsing.

For example, Abacha and al. [19] have a corpus based strategy close to [20] and this line of research. They aim at learning patterns from a list of seed terms corresponding to pairs of entities known to be in some target relations. Other works based on pattern matching as AliBaba [21-23], learn surface patterns using sequence alignment of sentences to derive “motifs”. This method is based on a list of terms that represent interactions. Only interaction patterns are learned and no new term to symbolize interaction can be discovered. With our method, linguistic patterns are automatically learned to detect interactions (interaction patterns) and also, at the same time, to characterize the interactions (characterization patterns). In addition, the terms and the patterns do not need to be provided. They are automatically extracted by the method. It thus provides new knowledge.

The key idea of our approach is to take advantage of an hybridization of data mining and NLP for Biological Natural Language Processing (BioNLP). Data mining techniques, such as extraction of frequent sequential patterns [24], enable the discovery of implicit, previously unknown, and potentially useful information from data [25]. Our contribution is an original method to automatically produce *patterns* (which can be seen as a kind of linguistic rules) from text collections.

The problem of data mining techniques is that, in general, too many patterns are generated. That is why, our method is based on recursive sequential pattern mining with constraints from the NLP field to tackle the discovery of gene interactions. The patterns output convey a model of the interactions that are enhanced with semantics information (modalities, biological contexts, interactions types).

Only limited resource is required: the text collection (used as a training corpus) which only contains sentences with interactions and where only gene names are tagged but not the interaction. In particular, terms and patterns are automatically discovered from texts without other resources.

To the best of our knowledge, there are few methods that extract the interactions and also provide associated semantics information on the extracted interactions thanks to the discovered patterns which are understable and can be manually modified by a human expert.

In addition, we propose to use background knowledge, a well-established biomedical corpora and a gene interaction database, in order to assess the relevance of patterns, used as linguistic rules, and to help an expert^a^ to select them. We describe a validation method based on the idea that the *relevant* rules convey information that must be consistent with the background knowledge. This method is interesting because the validation of rules is currently widely based on human checking which is highly time consuming. Last but not least, we conduct extensive experiments highlighting how our approach enables to discover interactions and their characterizations and we present a discussion of the results.

## Method

**Figure 1:**
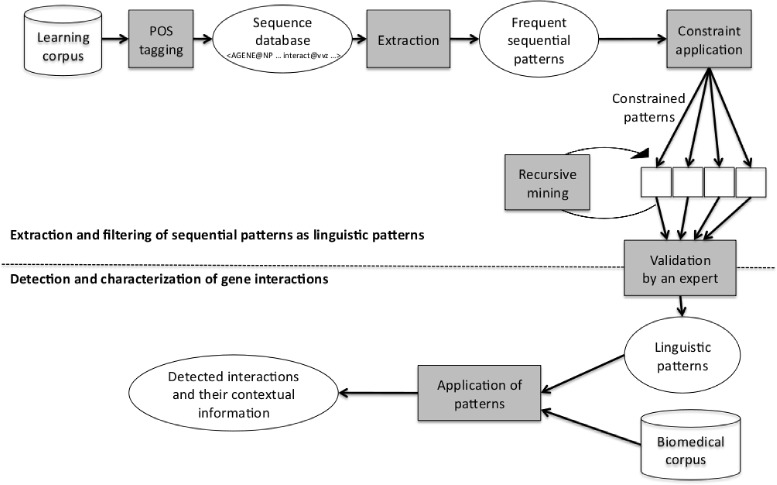
General framework to extract gene interactions. Figure 1 presents the overall process to detect and characterize gene interactions. There are two steps. The first step is the extraction of patterns. Sequential patterns are mined from a learning corpus that contains sentences representing gene interactions. In order to reduce the number of extracted patterns, constraints and recursive mining are applied. At the end, few sequential patterns corresponding to candidate linguistic interaction or characterization rules remain. A key point is that the sequence of words expressing the interaction in a pattern is automatically discovered. As an example, the sequence of words <*AGENE*, "association" > in the pattern “*AGENE* association with *AGENE*” (see Table 5) where a small gap may appear between *AGENE* and "association" are discovered by our method. This pattern conveys an interaction between two genes (denoted AGENE) in association. The sequential patterns are then analyzed and validated by experts. The ones that are not relevant for interaction detection or characterization are removed. The second step is the application of those validated patterns as linguistic rules to discover and characterize interactions in new corpora.

This section presents our method to produce linguistic rules in order to discover interactions and their characterizations. Figure 1 gives a global view of the process.

### Background: sequential pattern mining

Sequential pattern mining is a well-known technique introduced in [24] to find regularities in database of sequences, and for which there are several efficient algorithms (e.g. [26-30]). A *sequence* as used in our method is an ordered list 〈*i*_1_…*i*_*m*_〉, where the elements of the list *i*_1_…*i*_*m*_ are called items^b^.

A sequence *S*_1_=〈*i*_1_…*i*_*n*_〉 is *included* in a sequence *S*_2_=〈*i*1′…*im*′〉 if there exist integers 1≤*j*_1_<...<*j*_*n*_≤*m* such that *i*_1_=*ij*_1_′,..., *i*_*n*_=*ij*_*n*_′. The sequence *S*_1_ is called a *subsequence* of *S*_2_, and we note *S*_1_≼*S*_2_. For example, we have 〈*bd*〉≼〈*abcd*〉.

A sequence database *SDB* is a set of tuples (*sid*,*S*), where *sid* is a sequence identifier and *S* a sequence. For instance *SDB*_1_={(1,〈*abcd*〉), (2,〈*bde*〉), (3,〈*acde*〉), (4,〈*adcb*〉)} is a database of four sequences.

A tuple (*sid*,*S*)*contains* a sequence *S*_*α*_, if *S*_*α*_ is a subsequence of *S*. The *support* of a sequence *S*_*α*_ in a sequence database *SDB*, denoted *sup*(*S*_*α*_) is the number of tuples in the database containing *S*_*α*_. For example, in *SDB*_1_*sup*(〈*bd*〉) = 2, since sequences 1 and 2 contain 〈*bd*〉. Notice that for notational convenience, sometimes the *relative support* is used. In this case, the support *sup*(*S*_*α*_) is the relative number of tuples in the database that contain *S*_*α*_, $sup(S_{\alpha }) = \frac { |\{ (sid, S) ~|~ (sid, S) \in SDB \wedge (S_{\alpha } \preceq S) \}|}{|SDB|}$.

A *frequentsequential pattern* is a sequence such that its support is greater or equal to a given support threshold *minsup*.

### Extraction of sequential patterns in texts

For the extraction of sequential patterns from biological texts, we use a training corpus which is a set of sentences that contain interactions (but not annotated) and where the genes are identified. In this paper we consider sentences containing interactions and at least two gene names to avoid problems introduced by the anaphoric structures^c^ [31]. The training corpus, with tagged gene names, is selected by an expert. The items are combinations of lemma and POS tags^d^. POS tag information is important to disambiguate words (e.g., “*form*” the noun vs “*to form*” the verb). The sequences of the database are the interaction sentences where each word is replaced by the corresponding item. The order relation between items in a sequence is the order of words within the sentence. For example, let us consider two sentences that contain gene interactions: “Recent studies have suggested that c-myc may be vital for regulation of hTERT mRNA expression and telomerase activity”. and “Injection of frpHE mRNA in Xenopus embryos inhibited the Wnt-8 mediated dorsal axis duplication”. All gene names are replaced by a specific item, *AGENE*, and the other words are replaced by the combinations of their lemma and their POS tag. An excerpt of the database that contains the sequences associated to those two sentences is given in Table 1. The sequential patterns are extracted from this database.

**Table 1:** **Example of a sequence database**

**ID**	**Sequence**
...	...
S1	〈*R* *e* *c* *e* *n* *t* *@* *j* *j* *s* *t* *u* *d* *y* *@* *n* *n* *s* *h* *a* *v* *e* *@* *v* *h* *p* *s* *u* *g* *g* *e* *s* *t* *@* *v* *v* *n* *t* *h* *a* *t* *@* *i* *n*/*t* *h* *a* *t* *A* *G* *E* *N* *E* *m* *a* *y* *@* *m* *d* *b* *e* *@* *v* *b* *v* *i* *t* *a* *l* *@* *j* *j*
	*for@in regulation@nn of@in AGENE mrna@np expression@nn and@cc telomerase@nn*
	*activity@nn.@sent 〉*
S2	〈*i* *n* *j* *e* *c* *t* *i* *o* *n* *@* *n* *n* *o* *f* *@* *i* *n* *A* *G* *E* *N* *E* *m* *r* *n* *a* *@* *n* *p* *i* *n* *@* *i* *n* *x* *e* *n* *o* *p* *u* *s* *@* *n* *p* *e* *m* *b* *r* *y* *o* *@* *n* *n* *s* *i* *n* *h* *i* *b* *i* *t* *@* *v* *v* *d* *t* *h* *e* *@* *d* *t*
	*AGENE mediate@vvd dorsal@jj axis@nn duplication@nn.@sent 〉*
...	...

Table 1 shows an excerpt of a sequence database which contains two interaction sentences:

S1: *“Recent studies have suggested that c-myc may be vital for regulation of hTERT mRNA expression and telomerase activity.”* and

S2: *“Injection of frpHE mRNA in Xenopus embryos inhibited the Wnt-8 mediated dorsal axis duplication.”*.

The choice of a support threshold *minsup* is a well-known problem in data mining. With a high *minsup*, only few very general patterns can be extracted. With a low *minsup*, a lot of patterns can be found. Some interesting words, for example “interaction”, are not very frequent so that we set a low value of *minsup*. As a consequence, a huge set of patterns is discovered and it needs to be filtered in order to return only relevant patterns.

### Constraints and recursive mining

To reduce the number of extracted patterns, we use a combination of data mining methods. The constraint-based pattern paradigm (e.g., [32]) enables discovering patterns under user-defined constraints in order to drive the mining process towards the user objectives. Recursive mining [33] reduces the number of patterns by extracting their common structures.

#### Linguistic constraints

In pattern mining, constraints allow the user to define more precisely what should be considered as interesting. The most commonly used constraint is the constraint of frequency (*minsup*). However, it is possible to use different constraints [34]. In our method, in order to extract gene interaction patterns, we use three additional constraints.

The first constraint is that the pattern must contain two gene names, i.e. two *AGENE* items.

The second constraint is that the pattern must contain at least a verb or a noun.

Finally, among the patterns that satisfy the frequency and the two other previous constraints, we retain only the maximal ones with respect to the inclusion order ≼. That last constraint allows the redundancy between patterns to be reduced.

The constraints can be gathered in only one constraint $\mathcal {C}_{G}$ which is the conjunction of the three constraints. *SAT*(*C*_*G*_) is the set of patterns satisfying $\mathcal {C}_{G}$.

#### Recursive mining

Even if the new set of sequential patterns, $SAT(\mathcal {C}_{G})$, is significantly smaller than the initial set of all extracted sequential patterns without constraints, it can still be too large to be analyzed and validated by experts. To find a limited number of patterns corresponding to general structures among the whole pattern collection, we use the *recursive mining* technique of [33]. The key idea of this post-processing is to reduce the size of the output by successively repeating the mining process on the patterns themselves in order to extract the structure shared by the patterns. More precisely, at each step, the previous set of sequential patterns is used as a new sequential database, and a new extraction is made. The process stops when no more than *k* patterns are obtained by the extraction, where *k* is a parameter set by the user.

Our target is to identify at least one pattern by verb or noun that appears in the patterns in $SAT(\mathcal {C}_{G})$. So, for each verb or noun denoted *X*_*i*_, that appears in $SAT(\mathcal {C}_{G})$, we collect the set $E_{X_{i}}$ of patterns containing *X*_*i*_, $E_{X_{i}} = \{ s \in SAT(\mathcal {C}_{G}) ~|~ \langle X_{i} \rangle \preceq s \}$. Note that some frequent patterns can contain more than one noun and/or verb (so several *X*_*i*_). In this case, the pattern is duplicated in the $E_{X_{i}}$ of each noun and/or verb.

For a given value of *k*, we apply the recursive mining post-processing technique on each $E_{X_{i}}$. At each extraction step we select only the patterns that satisfy $\mathcal {C}_{G} $, and use a relative minimum support threshold $minsup = \frac {1}{k}$. That threshold value and the maximality constraint guarantee that recursive mining process terminates in finite steps as proved in [35].

At the end of this post-processing of all $E_{X_{i}}$, the number of sequential patterns cannot exceed *n*×*k* where *n* is the number of verbs and nouns occurring in $SAT(\mathcal {C}_{G})$.

### Selection and categorization of patterns

The sequential patterns are then analyzed and validated by experts. The ones that are not relevant for interaction detection or characterization are removed. The remaining ones are selected as linguistic extraction rules [36]. A selected pattern is classified with respect to the kind of information conveyed by the pattern. There are two main classes of patterns: *interaction patterns* and *characterization patterns*. The first class indicates what kind of interaction between genes is found (e.g., inhibition). The second class is *characterization patterns*. It is built by the experts and can be completed with other classes if other kinds of information extraction rules are found. There are two kinds of *characterization patterns*: *modality patterns* and *biological context patterns*. Examples of patterns are discussed in Section “Extracted sequential patterns”.

When the experts have validated and classified all patterns in the different categories, they are applied as linguistic rules to discover and characterize interactions in new corpora.

In practice, this step is not time consuming as shown in the following and can be helped by using background knowledge to support pattern validation as proposed in the Section “About validation of sequential patterns as linguistic extraction rules”. Detection with sequential patterns representing interactions, modalities or biological contexts is much more elaborated than just a co-occurrence detection. Indeed, the order of the words and the context are important, they provide semantics information. For instance, the sub-categorization of the verb given by the POS tagging indicates the passive or active verb and identifies the direction of the interaction. Prepositions can give this information when the pattern does not contain a verb, for example: 〈*activation@nn of@in AGENE by@in AGENE* 〉.

Note the genericity of the approach, indeed the extracted patterns allow genetic interactions to be discovered as well as physical protein interactions.

## Results

In this section, we present the experiments and results. First, the training corpus is detailled. Then, the sequential pattern extraction is described. Finally, the results of the application of the extracted patterns on testing corpora are presented.

### Training corpus

Genes can interact with each other through the proteins they synthesize. Moreover, although there are conventions, the same word can represent a gene name and the protein synthesized by the gene. Biologists know from the context if the sentence is about protein or gene. To discover the linguistic patterns of interactions between genes, we merge two different corpora containing genes and proteins, to create the training corpus. The first corpus contains sentences from PubMed abstracts, selected by Christine Brun^e^ as sentences containing gene interactions. It contains 1,806 sentences. That corpus is available as a secondary data source for the learning tasks “Protein-Protein Interaction Task (Interaction Award Sub-task, ISS)” from BioCreAtIvE Challenge II [8]. The second corpus [37] contains 2,995 sentences mentionning interactions between genes selected by an expert. The union of those two corpora results in a dataset containing 4,801 sentences about gene interactions.

### Sequential pattern extraction

#### Data mining task

As previously mentionned, the extraction of sequential patterns from the training corpus needs the computation of POS tags. For this task, we use the *treetagger* tool [38].

In addition, for the data mining task, *minsup* is set to 10. It means that a sequential pattern is frequent if it appears in at least 10 sentences (i.e. in more than 0.2% of sentences). Indeed, with that threshold some irrelevant patterns are not taken into account while many patterns of true gene interactions are discovered. Note that other experiments, not reported here, have been conducted with greater *minsup* values (15 and 20). With those greater *minsup*, some relevant patterns for interaction detection are lost.

More than 32 million of frequent sequential patterns are extracted, with *minsup* equals to 10. This number is large but the extraction takes only 15 minutes (the extraction tool is *dmt*4*sp* [39]). The application of constraints significantly reduces the number of sequential patterns. Indeed, the number of sequential patterns satisfying the constraints is about 65,000. Note that the application of the constraints was not time consuming and takes less than two minutes. However, the number of remaining patterns is still prohibitive for analysis and validation by human experts.

The recursive mining also reduces significantly the number of sequential patterns. From the extracted patterns, we build a subset of patterns for each noun or verb. The number of built subsets is 515 (365 for nouns, 150 for verbs). The recursive mining of each subset exhibits at most *k* sequential patterns to represent that subset. In this experiment, we set the parameter *k* to 4. It allows several patterns to be kept for each noun or verb in order to cover a sufficient number of different cases (for example 4 patterns corresponding to 4 syntactic constructions with the verb *inhibit@vvn* are computed). At the end of the recursive mining, there remain 667 sequential patterns that can represent interactions or their characterizations^f^. That number, which is significantly smaller than the previous one, guarantees the feasibility of an analysis of those patterns by experts. The recursive mining of those subsets is also very fast and takes about 2 minutes.

#### Extracted sequential patterns

The 667 remaining sequential patterns were analyzed by two experts in 90 minutes.

The patterns are grouped together by noun or verb, the experts have thus to classify 380 groups. But some nouns or verbs are repeated with different POS tagged information (e.g., *analyze@vvd* and *analyze@vvn*), so these groups are not considered independently by the experts and it helps the validation task (for instance both versions of verb "analyze"İ are pruned together). Actually, there are 285 different nouns and verbs. Moreover, at this point of the validation, the patterns are roughly split into three sets by the experts: "interaction patterns", "characterization patterns"İ and "not relevant".

Finally, the experts validated 232 sequential patterns for interaction detection, 231 patterns for characterization of interactions and they removed the remaining (i.e. 204 unuseful patterns). Indeed, the latter do not convey information about interactions, in particular there are generic verbs like "appear"İ and "contain". Among the first group of 232 patterns, some explicitly give the type of the interactions. For example, 〈*AGENE interact@vvz with@in AGENE* 〉, 〈*AGENE bind@vvz to@to AGENE* 〉, 〈*AGENE deplete@vvn AGENE* 〉 and 〈*activation@nn of@in AGENE by@in AGENE* 〉 describe well-known interactions (binding, inhibition, activation). Note that when the patterns are applied, zero or several words may appear between two consecutive items of the pattern. For example, the pattern 〈*AGENE interact@vvz with@in AGENE* 〉 matches the sentence “ <gene_name=MYC > interacts with <gene_name=STAT3 >”. and also the sentence “ <gene_name=MYC > interacts with genes in particular <gene_name=STAT3 >”^g^.

Other patterns represent more general interactions and express the fact that a gene plays a role in an activity of another one. Representative patterns of this kind are for instance 〈*AGENE involve@vvn in@in AGENE* 〉, 〈*AGENE play@vvz role@nn in@in the@dt AGENE* 〉 and 〈AGENE play@vvz role@nn in@in of@in AGENE 〉. Note that the *“involve”* verb and the *“play role in”* phrase were not reported in [40,41] and [21].

**Figure 2:**
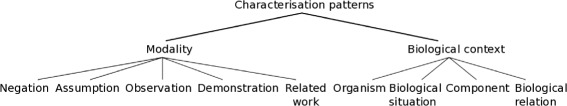
Taxonomy for characterization patterns. Figure 2 describes the taxonomy used to classified the extracted sequential patterns.

The second group of 231 patterns for characterization represents other kinds of semantics information: modalities or biological context, for instance, 〈*in@in fibroblast@nns AGENE AGENE* 〉 or 〈*the@dt possibility@nn that@in/that AGENE AGENE* 〉. Figure 2 depicts the taxonomy that we define and use in our experiments for the characterization patterns. That taxonomy was built with the help of the extracted patterns. The *modality patterns* express the confidence in the detected interactions. Modality can be seen as a kind of uncertainty [42]. We define four levels of confidence: *Assumption*, *Observation*, *Demonstration* and *Related work*, and another subclass representing the *Negation* (patterns denoting evidence of absence of interaction). For example, the sentence "It suggests that <gene_name=MYC > interacts with <gene_name=STAT3 >" has a lower confidence than "It was demonstrated that <gene_name=MYC > interacts with <gene_name =STAT3 >". The *biological context patterns* indicate information about the biological context of interactions, for example the disease or the organism involved in the interaction. That class is split into four subclasses: *organism*, *component*, *biological situation* and *biological relation*. The subclass *organism* represents the organisms involved in the interaction (e.g., “mouse”, “human”). The subclass *component* represents the anatomy/biological components (e.g. “breast” or “fibroblast”). The subclass *biological situation* gives the framework of interactions, for example, “cancer”, “tumor” or “in vitro”. The last subclass gives, when applicable, the *biological relation* (e.g., “homology”).

The sequential patterns obtained are linguistic extraction rules that can be used on biomedical texts to detect and characterize interactions between genes. Note that to be applied, those patterns do not need a full syntactic analysis of a sentence.

Indeed, the matching process tries to instantiate each element of the pattern in the given sentence. For each pattern, every possible matching within the sentence is tested and not only the first one.

### Application: detection and characterization of gene interactions

We have evaluated the quality of the sequential patterns found in the previous section as information extraction rules. In this section, we present the experimental settings and the results.

#### Testing corpora and evaluation criteria

##### Testing Corpora

We have considered three well-known testing datasets (cf Table 2 and Table 3): *AIMed* [43], *BioInfer* [44], *HPRD50* [45] and a fourth testing corpus extracted from PubMed [1] (more information is given in the next section). Note that, in *AIMed*, *BioInfer* and *HPRD50*, the names of genes are already identified and tagged. More information about those corpora can be found in [46].

**Table 2:** **Results of the application of the extracted patterns**

**Corpus**	**#**	**Recall**	**Precision**	***f*** **−** ***s*** ***c*** ***o*** ***r*** ***e***	***f*** **−** ***s*** ***c*** ***o*** ***r*** ***e***
	**Sentences**				**presented in [** 11 **]**
					**details given in Table** 3
*AIMed*	1955	78.6	35.6	**49**	[34.7,41.5]
*BioInfer*	1100	46.5	25.3	32.8	[15.9,40.6]
*PubMed*	200	75.0	83.0	78.7	−
*HPRD50*	145	66.8	46.7	55.0	[38.3,69.8]

Table 2 gives the list of the four testing corpora used to evaluate the proposed approach, and the results of the evaluation. The meaning of the columns is: the name of the corpus, the number of the sentences in the corpus, the recall score of the proposed approach applied on the corpus, the precision score of the proposed approach applied on the corpus, the*f*-*score* of the proposed approach applied on the corpus. The last column indicates the range of the *f*-*scores* presented in [11] with also a cross-corpus validation.

**Table 3:** **Details of information presented in paper [** 11 **]**

**Method**	**SL**	**SL**	**SpT**	**SpT**	**kBSPS**	**kBSPS**	**edit**	**edit**	**APG**	**APG**
**Training corpus**	**(AIMed)**	**(BioInfer)**	**(AIMed)**	**(BioInfer)**	**(AIMed)**	**(BioInfer)**	**(AIMed)**	**(BioInfer)**	**(AIMed)**	**(BioInfer)**
*AIMed*	-	41.5	-	34.7	-	40.3	-	39.6	-	37.9
*BioInfer*	40.6	-	24.3	-	24.8	-	15.9	-	22.5	-
*HPRD50*	59.0	61.8	43.2	51.3	51.0	69.8	38.3	62.4	61.6	62.1

The acronyms used in this table are the ones used in paper [11]: SL: Shallow linguistic kernel; SpT: Spectrum tree kernel; kBSPS: k-band shortest path spectrum kernel; edit: Edit distance kernel; APG: All-paths graph kernel. See paper [11] for more details.

##### Construction of the PubMed corpus

In order to test the sequential patterns extracted in the previous section as linguistic extraction rules to characterize interactions, we need a testing corpus.

We have built a testing corpus that is a subset of abstracts from the PubMed database. It is built in two steps which are described below. The first step is the selection of abstracts from PubMed. In the PubMed database, each paper has an identifier called PMID (PubMed IDentifier). For each official acronym of gene in the HUGO [47] dictionary, a request is sent to PubMed in order to get all PMID of papers that contain the gene. An index of genes and their associated papers is thus created. Then the inverted index is computed, i.e. the index that associates to each PMID the list of genes. From that second index, the PMIDs that do not have at least two gene names in their list are pruned. Indeed, as we are looking for interactions between genes, it implies that at least two genes are mentioned in the text. There remains 624,519 PMIDs. The second step is the named-entity recognition. Sometimes, the gene name used to index an abstract and the gene name that appears in the abstract text are different. Indeed, a gene can be represented by different synonymous forms. It is thus important to identify the gene in the text; that task is called Named-Entity Recognition (NER). We propose to use a "dictionary-based" approach [48]. Although that kind of approaches usually has a good precision, it does not provide a good recall. We propose some improvements to increase the recall.

First, all genes associated to the PMID of an abstract are searched into that abstract using official acronyms from the HUGO dictionary. With that approach only 48.1% of abstracts have at least two recognized genes. In addition, we identified 182 official acronyms as common English words (e.g., AGO, AS, BAD)^h^. In order to reduce the number of mistakes, they are considered as gene names only when they are in uppercase.

Second, in order to improve the number of recognized genes in abstracts, other fields of the HUGO dictionary are used: old acronyms, alias acronyms, and complete names. With that improvement, 61.7% of abstracts have at least two recognized genes. Note that this improvement is mainly due to the alias acronyms (+ 9%).

The last improvement is the use of significant parts of the complete official name. The official name is often long, and authors do not write it completely. Instead of looking for the complete name, we look for significant parts of it. We identify three common kinds of significant parts: a word ending by “in” (e.g., “insulin”), a word ending by “ase” (e.g., “transferase”) and a word followed by “protein” (e.g., “AE binding protein 1”). For instance, for gene “alkaline ceramidase 3”, the significant part is “ceramidase” and thus to recognize this gene name in texts, only “ceramidase” would be used. The plural forms are also taken into account (e.g., “caspases”, “kinases”). With that improvement, 66.1% of the 624,519 abstracts have at least two recognized genes and form the testing corpus.

##### Evaluation criteria for the extraction of gene interactions and their contextual information

We evaluate our approach with a cross-corpus evaluation to show the genericity of the proposed approach. It means that we extract the patterns from a corpus and apply them on the other four corpora [11]. Note that in the literature many approaches are evaluated with a cross-validation, which means that a corpus is split in several parts, one part is used to learn and the rest is used to apply.

It is thus much more difficult to get good results with a cross-corpus evaluation than a cross-validation.

Indeed, there is more heterogeneity between corpora (i.e., corpus characteristics are different) than between parts of a single corpus [11].

We use the *f*-*score* function as an evaluation measure, which is defined as *f*-$score = \frac {2\times Precision \times Recall}{Precision + Recall}$.

#### Detection of gene interactions

We have applied the 232 extracted sequential patterns as linguistic extraction rules to detect interactions on the four corpora.

All corpora used for evaluation have all gene names readily tagged. This means that our results only measure the performance of gene interaction extraction and are not influenced by the issue of named entity recognition. Therefore, to compute the f-score, a true positive is a couple of mentioned gene names in the sentence (i.e. the gene names given in the tags) which are in interaction and detected as an interaction by our method. Table 2 gives the results. We did not have any gold standard reference to evaluate the results for the testing corpus from PubMed. Since we cannot implement an automatic validation, we randomly took 200 sentences among the sentences of the PubMed testing corpus. Then, we carried out a POS tagging and assessed the performances of the extraction rules to detect interactions in the 200 sentences^i^. The *f*-*score* for the gene interaction detection for the testing corpus is 78.7. In Table 2, the last column indicates the range of the *f*-*scores* presented in [11] with a cross-corpus validation. Several kernel-based approaches are presented in [11], the range allows to show the worst and the best results among all those methods. Note that the best result of the ranges is not achieved in practice by the same method. Our approach gives results comparable to the results given by state-of-the-art methods and it is even better for the gene interaction detection in AIMed. This last result is important because AIMed is the largest corpus and the most commonly used in the literature. Moreover, our approach is simple and allows more information that just the presence of an interaction to be extracted. Indeed, thanks to the patterns, semantics information can also be extracted, contextual information (see next section) but also information about the kind of interaction (e.g., inhibition, binding) and the direction of the interaction.

#### Characterization of gene interaction

The method also gives information about modality and about the biological context: biological situation, component, organism, biological relation. For that characterization task, there exist some methods dealing with the subtask of the detection of sentences containing uncertainty [42] (modality can be seen as a kind of uncertainty) but few adress the biological characterization problem. It was thus difficult to compare our result for the interaction characterization with a gold standard. We randomly took 200 sentences containing at least two gene names among the sentences of the testing corpus extracted from PubMed. Those sentences are not the same ones that are used to evaluate the interaction detection but they come from the same testing corpus PubMed. Out of 200 interactions, there are 149 characterizations (71 modalities and 78 biological context). The sentences have been annotated by a computer scientist with specialisation in NLP and a biologist. Then, we evaluated the precision and recall. The characterization patterns are applied on a pair of genes that is already detected as in interaction. We evaluate the characterization at the interaction level. The precision is 88% and the recall is 69% (*f*-*score* =77). Several reasons explain why the recall is not greater and are discussed in the next section.

## Discussion

In this section, the results of the previous section are discussed from a qualitative point of view and we present a process to support pattern validation.

### About interaction detection

In the experiments a linguistic pattern is matched against a whole sentence at a time. That wide scope may introduce ambiguities in the detection of interactions, and false positives, when more than two genes appear in a sentence. For example, in sentence *“FGF-7 recognizes one FGFR isoform known as the FGFR2 IIIb isoform or keratinocyte growth factor receptor (KGFR), whereas FGF-2 binds well to FGFR1, FGFR2, and FGFR4 but interacts poorly with KGFR”.* an interaction between FGFR2 IIIb and FGFR1 is detected. Actually, there is no interaction between those two genes, they only appear in two different propositions of the same sentence. FGFR1 interacts with FGF-2 in the second proposition but since there is no limitation of the scope, an interaction between FGFR1 and FGFR2 IIIb is also detected. Several cases are possible: when several binary interactions are present in the sentence or when the interaction is n-ary (*n*≥3). The case of n-ary interactions can be solved with a training data set containing n-ary interactions. The other cases can be treated by introducing limitations of pattern scope, for example cue-phrases (e.g., *but*, *however*).

False negatives depend on the absence of some nouns or verbs of interaction in the patterns. For example, the noun “modulation” is not discovered whereas the verb “modulate” appears in sequential patterns. This suggests that the use of linguistic resources (e.g. lexicon or dictionary), manually or semi-automatically, would improve interaction patterns and thus interaction detection.

### About interaction characterization

The false negatives, which are dependent on the absence of some patterns, are also an important problem for interaction characterization.

For example, in our experiments in the sentence *“ <gene_name=BRCA1 > interacts in vivo and in vitro with the Rb-binding proteins, <gene_name=RBBP7 > and <gene_name=RBBP4 >[...]”* the biological situation “in vitro” is detected whereas “in vivo” is not detected. Indeed, there is no sequential pattern extracted from the training corpus that contains “in vivo”. That case is considered as true positive for in vitro interaction and as false negative for in vivo interaction. The recall (69%) is strongly dependent on the number of false negatives. Note that the false negatives mainly come from biological contexts not sufficiently represented (about 92%). It is explained by the difficulty to have a training corpus that contains all biological context (e.g, body parts as “liver”, “pituitary gland”, diseases). As for interaction detection, using a specialized lexicon would increase the vocabulary and thus the number of patterns and would improve those results.

### About validation of sequential patterns as linguistic extraction rules

Section “Method” shows how the sequential patterns are automatically extracted from a corpus. Those patterns are then analyzed and validated by two experts as linguistic extraction rules. But sometimes, the needed resources (e.g., time, expert) can be missing or the number of sequential patterns can be too large to be easily managed by a human. In those cases, for the selection and validation of patterns, we propose an automatic process based on the use of background knowledge. The selection is thus less accurate than a manual selection but can be automatic.

The automatic validation process is based on two steps.

First, each sequential pattern is applied on a corpus called *rule validation corpus*. It provides for each pattern the following information: the genes detected as interacting and the associated sentences.

Second, a gene interaction database is used as an *oracle* to assess the patterns. In our method, the rule validation corpus comes from the PubMed papers and the gene interaction database is BioGRID. Our idea is that the relevant patterns, when applied on the validation corpus, retrieve interactions that must be consistent with the gene interaction database. An interaction detected by a sequential pattern is considered as a false positive if the interaction does not exist in the gene interaction database, else it is a true positive (same gene names and same PMID)^j^.

A pattern with a high number of true positives is likely to be interesting.

Table 4 gives an excerpt of the information provided for each pattern. It contains the number of interactions detected by the pattern and the number of detected interactions that are correct with respect to the oracle, i.e. interactions that also exist in BioGRID. For example, the fifth pattern can be read as “a *gene* followed by the verb *bind* in present tense, then by the word *to* and a gene name”. This pattern detects 8 interactions and 5 of them are in BioGRID. For example, it detects that the following complex sentence “Cbl is a cytosolic protein that is rapidly tyrosine phosphorylated in response to Fc receptor activation and binds to the adaptor proteins Grb2, CrkL, and Nck”. contains an association between two signalling molecules (CBL and GRB2).

**Table 4:** **Examples of information about the application of information extraction rules**

	**Number of**	**Number of**
**Information**	**retrieved interactions**	**true positives**
**extraction rule**		
*AGENE AGENE* the@dt	6	1
response@nn		
*AGENE AGENE* serine@nn	3	3
*AGENE* reveal@vvd *AGENE*	0	undefined
*AGENE* association@nn	6	4
with@in *AGENE*		
*AGENE* bind@vvz to@to *AGENE*	8	5

Table 4 gives an excerpt of provided information about patterns extracted from the PubMed corpus. The meaning of the columns is: sequential pattern, number of interactions detected by the pattern and number of detected interactions that are correct with respect to the oracle, i.e. interactions that also exist in BioGRID.

The first pattern can be read as “a *gene* followed by a *gene* then by the word *the* and the word *response*”. This pattern detects 6 interactions and 1 is in BioGRID. The second pattern can be read as “a *gene* followed by a *gene* then by the word *serine*”. It detects 3 interactions that are all in BioGRID. The third pattern can be read as “a *gene* followed by the verb *reveal* in past tense, then by a *gene*”. This pattern does not detect interactions in the rule validation corpus, thus no information is provided to evaluate it. The fourth pattern can be read as “a *gene* followed by the noun *association*, then by the word *with* and a gene name”. It detects 6 interactions out of which 4 are in BioGRID. The fifth pattern can be read as “a *gene* followed by the verb *bind* in present tense, then by the word *to* and a gene name”. This pattern detects 8 interactions and 5 of them are in BioGRID. For example, it detects that the following complex sentence “Cbl is a cytosolic protein that is rapidly tyrosine phosphorylated in response to Fc receptor activation and binds to the adaptor proteins Grb2, CrkL, and Nck.” contains an association between two signalling molecules (Cbl and Grb2).

Those measures can be used to automatically select patterns as linguistic information extraction rules.

To end up with a more speculative note, this step could also be interesting even when there is an expert to select the patterns by providing more information to help them. In addition, a pattern with low number of true positives can retrieve sentences that really contain interactions. This can be the case if the interaction is not reported in BioGRID or is reported but the gene names in the sentence are other gene names than the ones used in BioGRID. Therefore, it is interesting to provide, for each pattern, the detected interaction sentences. Table 5 gives an excerpt of interactions detected by the sequential pattern: “*AGENE* association with *AGENE*”. For instance, the pattern detects that in the paper with PMID 10204582, an interaction between genes SHC1 and CRKL is mentioned (the pattern matches a sentence of the abstract) but according to BioGRID, there is no interaction between SHC1 and CRKL. The discovered interaction in the sentences in paper 10204582 is thus unexpected in BioGRID. The pattern also detects that in this paper, an interaction between genes CBL and CRKL is mentioned, and indeed, according to BioGRID, there is an interaction between CBL and CRKL mentioned in paper 10204582. It is interesting to note that the three genes involved harbour all three similar biological functions (they are all signalling molecules) and that their association is fully relevant as exemplified by the strong functional connectivity detected in the STRING database between those three genes. Therefore the extracted interaction between genes SHC1 and CRKL is fully relevant even if it does not appear in BioGRID. Of course, more systematic studies should be undertaken to ascertain this, but this is beyond the scope of the present paper.

**Table 5:** **Example of information for a sequential pattern**

**PMID**	**Gene 1**	**Gene 2**	**BioGRID verdict**	**Sentence**
10204582	SHC1	CRKL	not in BioGRID	These results suggest a fundamental role for the
				tyrosine phosphorylation of Cbl, CrkL, SLP-76, and
				**<gene_name="SHC1" >** and the **association**
				of Cbl **with** **<gene_name="CRKL" >**, SLP-76,
				and Nck in Fc gammaRI signaling in human
				macrophages.
10204582	CBL	CRKL	in BioGRID	PP1, a specific inhibitor of Src kinases, inhibited
				the Fc gammaRI-induced respiratory burst,
				as well as the tyrosine phosphorylation of
				**<gene_name="CBL" >** and its inducible
				**association with** **<gene_name="CRKL" >**.

Table 5 gives 2 interactions (highlighted in bold in the table) detected by the sequential pattern: “*AGENE* association with *AGENE*”.

The meaning of the columns is: the id number of the paper in PubMed, the genes that interact, the verdict of the oracle and the sentence where the interaction is recognized. For instance, the pattern detects that in paper 10204582, an interaction between genes SHC1 and CRKL is mentioned, because the pattern matches a sentence of the abstract of the paper, but according to BioGRID, there is no interaction between SHC1 and CRKL and the discovered interaction in the sentences in paper 10204582 is unexpected because not in BioGRID and interesting. The pattern also detects that in this paper, an interaction between genes CBL and CRKL is mentioned, and indeed, according to BioGRID, there is an interaction between CBL and CRKL mentioned in paper 10204582.

## Conclusions

We have proposed an original approach to help experts to design linguistic information extraction rules by automatically extract sequential patterns filtered by linguistic constraints and recursive mining. Unlike existing methods, our approach is independent of syntactic parsing and only requires the training corpus as external resource to learn patterns (note that interaction clues are not annotated in the training corpus). The patterns representing interactions and their characterizations are automatically discovered from texts. An advantage of the use of sequential patterns as linguistic rules is that they are understandable and manageable by an expert. If needed, the expert can easily modify the proposed rules or add new ones. To the best of our knowledge, there are few methods that automatically extract interaction patterns and also characterization patterns (i.e., patterns for contextual information about the discovered interactions).

The experiments show how our approach enables to discover interactions and their characterizations. Our approach gives results comparable to the results given by state-of-the-art methods and is even better for the gene interaction detection in AIMed. The main advantages of our approach are that, first, semantics information are extracted in addition to the information about the presence of an interaction; second, the patterns, used as extraction linguistic rules, are automatically discovered. Further work will look how enhance the extracted patterns thanks to other information sources (e.g., specialized dictionaries).

## Availability of software and supporting data

The extracted gene interactions from PubMed are available at https://bingotexte.greyc.fr/. The evaluation corpora from PubMed are available at https://cremilleux.users.greyc.fr/jbms/. The software (SMBio) that allows sequential patterns of gene interactions to be extracted is available at https://bingo2.greyc.fr/?q=node/22. The list of the 182 official acronyms identified as common English words is available at: https://bingotexte.greyc.fr/ambig_names.

## Endnotes

^a^ In the rest of the paper, the term ”expert” is used for a linguist or a biologist; both skills are useful to validate rules.

^b^ Notice that this is a simplified form of sequences, while in the general sequential pattern mining framework, a sequence is a list of *sets* of items, and not only a list of items.

^c^ An anaphoric structure is the use of a linguistic unit, such as a pronoun, to refer back to a gene name.

^d^ POS (Part-Of-Speech) tags are grammatical information about words. For example, *nn* means common noun and *vvp* means verb in non-3rd personal singular present. The exhaustive list of POS tags can be found at: http://www.cis.uni-muenchen.de/~schmid/tools/TreeTagger/.

^e^*Institut de Biologie du Développement de Marseille-Luminy*.

^f^ The maximum number of patterns per verb and noun is 4, thus the maximum number of patterns after applying recursive mining is 2060. The number of patterns in the results is only 667 because some verbs and nouns are represented by less than 4 patterns.

^g^ Note that such a functional relationship between MYC and STAT-3 can be illustrated by the fact that the expression of the c-myc gene is under the control of the STAT-3 signalling pathway (see [49] for a review).

^h^ The list is available at: https://bingotexte.greyc.fr/ambig_names.

^i^ Discovered interactions for the whole testing corpus are available at https://bingotexte.greyc.fr/.

^j^ BioGRID provides information about gene interactions and the PMID of the articles where the interactions are mentioned. In order to measure the accuracy of the patterns, we take into account these two pieces of information.

## Abbreviations

NLP: Natural language processing
NER: Named-entity recognition
IE: Information exctraction
BioNLP: Biological natural language processing
POS: Part-Of-Speech
minsup: support threshold
PMID: PubMed IDentifier
